# N6-methyladenosine-related lncRNAs is a potential marker for predicting prognosis and immunotherapy in ovarian cancer

**DOI:** 10.1186/s41065-022-00222-3

**Published:** 2022-03-18

**Authors:** Xin Nie, Jichun Tan

**Affiliations:** 1 Center of Reproductive Medicine, Shengjing Hospital of China Medical University, Shenyang, China; 2 Key Laboratory of Reproductive Dysfunction Diseases and Fertility Remodeling of Liaoning Province, Shenyang, China

**Keywords:** m6A, lncRNA, Prognostic signatures, Ovarian cancer, Immunotherapy

## Abstract

**Background:**

With a lack of specific symptoms, ovarian cancer (OV) is often diagnosed at an advanced stage. This coupled with inadequate prognostic indicators and treatments with limited therapeutic effect make OV the deadliest type of gynecological tumor. Recent research indicates that N6-methyladenosine (m6A) and long-chain non-coding RNA (lncRNA) play important roles in the prognosis of OV and the efficacy of immunotherapy.

**Results:**

Using the Cancer Genome Atlas (TCGA) OV-related data set and the expression profiles of 21 m6A-related genes, we identified two m6A subtypes, and the differentially expressed genes between the two. Based on the differentially expressed lncRNAs in the two m6A subtypes and the lncRNAs co-expressed with the 21 m6A-related genes, single-factor cox and LASSO regression were used to further isolate the 13 major lncRNAs. Finally, multi-factor cox regression was used to construct a m6A-related lncRNA risk score model for OV, with good performance in patient prognosis. Using risk score, OV tumor samples are divided into with high- and low-score groups. We explored the differences in clinical characteristics, tumor mutational burden, and tumor immune cell infiltration between the two groups, and evaluated the risk score’s ability to predict the benefit of immunotherapy.

**Conclusion:**

Our m6A-based lncRNA risk model could be used to predict the prognosis and immunotherapy response of future OV patients.

**Supplementary Information:**

The online version contains supplementary material available at 10.1186/s41065-022-00222-3.

## Background

Ovarian cancer lacks specific symptoms, so about 75% of patients are already at an advanced stage upon diagnosis, consequently the five-year survival rate for patients is only 46% [1]. Currently, surgery in combination with radiotherapy and chemotherapy is the main treatment method, but it has limited therapeutic effect [2]. Moreover, current tumor markers and tumor stage systems are still ineffective at predicting survival outcome and therapeutic efficacy of heterogeneous ovarian cancer. An understanding of the genomic characteristics of ovarian cancer could help predict individual survival, recurrence risk, and treatment efficacy.

N6-methyladenosine (m6A) is the most important and abundant internal modification in not only messenger RNA (mRNA), but also lncRNA in higher eukaryotes. m6A affects various stages of RNA metabolism, folding, splicing, translation and degradation [3]. m6A interacts with m6A methyltransferase (writers), such as WTAP, METTL3, METTL14, RBM15, and ZC3H13, to add methyl groups to RNA. Special proteins that can bind to methylation binding sites (readers), such as YTHDC1, YTHDC2, YTHDF1, YTHDF2, YTHDF3, and HNRNPC, recognize the modified RNA and produce different functions. Relying on the role of m6A demethylase (erasers) like FTO and ALKBH5, the methylation modification process is dynamically reversible and plays a role in regulating the expression of various genes[4–7].Genetic changes and mutations of m6A regulators are related to a variety of diseases, including cell death and proliferation disorders, impaired self-renewability, developmental defects, abnormal immune regulation, and malignant tumor progression [8–13]. lncRNAs are a group of RNAs that are longer than 200 nucleotides without any protein coding potential[14], but are a newly discovered regulatory factor for gene expression and a variety of physiological and pathological processes [15–17]. m6A modifications in mRNA and lncRNA can predict the prognosis and therapeutic effect for a variety of tumors [18–21]. However, there are few studies specifically exploring m6A methylation in ovarian cancer.

In this study, using the Cancer Genome Atlas (TCGA) OV-related data set, we determined the prognosis of 21 m6A modifications in ovarian cancer, and used the expression profiles of m6A-related genes to identify two m6A subtypes. We then constructed an ovarian cancer risk model based on the differentially expressed lncRNAs between m6A subtypes and the lncRNAs co-expressed with m6A-related genes. This risk model was used to not only evaluate the predictive ability of the risk score for tumor prognosis, but also the benefit of existing immunotherapies and the development of new, more precise immunotherapy.

## Materials and methods

### Obtaining expression profile data and clinical information

**Fig. 1:**
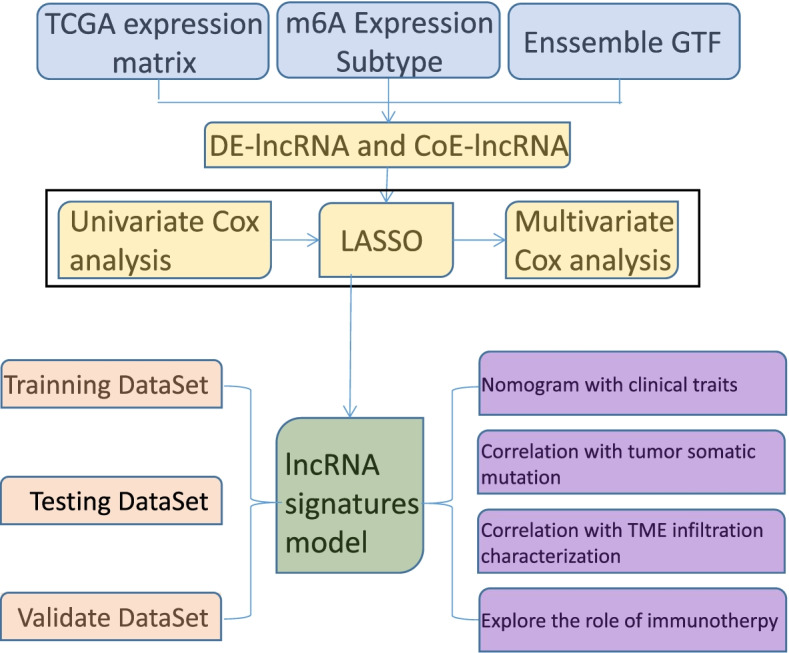
The work flow chart

The overall analysis progression is shown in the analysis flow chart (Fig. 1). First, we downloaded the OV expression profile data and clinical follow-up information data from the TCGA database (https://portal.gdc.cancer.gov/). Next, we determined the RNA-Seq data for the TCGA-OV samples by (1) removing all samples without clinical follow-up information, then (2) removing all samples with unknown survival time, < 30 days, and no survival status. Finally, we (3) converted the probe to gene symbols and (4) removed individual probes that corresponded to multiple genes, then (5) the median value was the expression of multiple gene symbols. After this pre-processing of the TCGA-OV data, there were a total of 323 tumor samples, with their clinical statistics listed in Table 1. Two eligible data from Gene Expression Omnibus (GEO) database (GSE26193 and GSE9891) were downloaded and an averaging method with the affy and simpleaffy packages was used to perform background adjustment and quantile normalization.

**Table 1:** Clinical characteristics of patients in TCGA-OV data set

Features		TCGA-OV
Survial		
	Status_0	120
	Status_1	203
Age		
	Age > 60	145
	Age < = 60	178
Gender		
	Female	323
Stage		
	Stage_II	14
	Stage_III	260
	Stage_IV	49
Grade		
	G1/2	44
	G3/4	279

### Consistent clustering of tumor m6A-related gene expression profiles

Using the ConsensuClusterPlus package in R software, unsupervised clustering was performed, and repeated 1000 times to ensure stable classification. The overall survival (OS) between different clusters was calculated using the Kaplan–Meier method.

### Differentially expressed genes between tumor m6A subtypes

Using the expression of m6A-related genes and the consistent clustering results, tumor samples were divided into two groups: m6A-1 and m6A-2. The limma package of R software analyzed the differential expression of genes between the two groups. We set the screening threshold to adjusted *P* < 0.05 and |log2 (Fold Change)|> 1, and used the genome annotation file (*.GTF) in Ensemble to extract the lncRNA of the differentially expressed genes (DEGs).

### Dimensionality reduction of gene features and construction of m6A-related lncRNA risk score model

Using the m6A subtype-related lncRNAs we constructed a risk score model for OV. First, we used the single-factor cox algorithm to remove redundant genes and reduce the size of the lncRNA gene set related to immune cell infiltration subtypes. Following reduction, the least absolute shrinkage and selection operator (LASSO) (Tibshirani 1996) method was used to filter the variables and further reduce the number of genes included the risk model. Finally, multi-factor cox regression was used to construct the risk score model for tumor immune cell infiltration. The calculation formula is as follows:

$$\mathrm{Risk }\_ scores= \sum Coef {\left(i\right)}^{* }Exp(i)$$

### Gene set enrichment analysis

To determine the influence of synergistic genetic changes on phenotypic changes, one or more functional gene sets in MSIGDB (Molecular Signatures Database) were selected for analysis (*.GMT), and then sorted based on the degree of correlation between gene expression data and phenotype, or the amount of expression. Then, gene set enrichment analysis (GSEA) (2005) with both gene ontology (GO) and Kyoto Encyclopedia of Genes and Genomes (KEGG) was used to determine whether those genes were enriched in the upper or lower part of the gene list.

### Statistical analysis and hypothesis testing

All the statistical comparisons involved in this study and the hypothesis testing for the significance of differences between groups were conducted in R 3.6 (http:// www.R-project.org). In all analyses, *P*-values were bilateral, and *P* < 0.05 was considered statistically significant.

## Results

### Molecular characteristics of m6A-related genes in OV

**Fig. 2:**
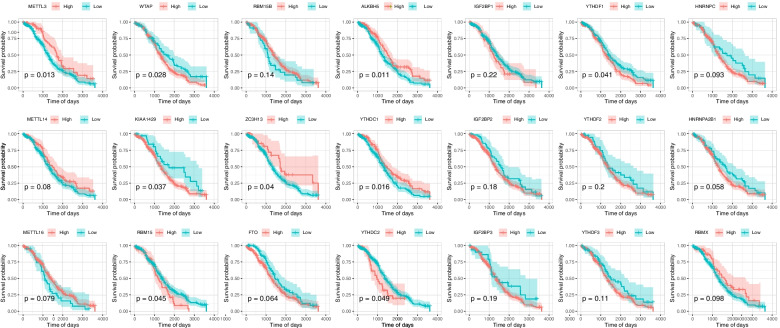
The expression and survival analysis of the 21 m6A-related genes in the TCGA-OV dataset

**Fig. 3:**
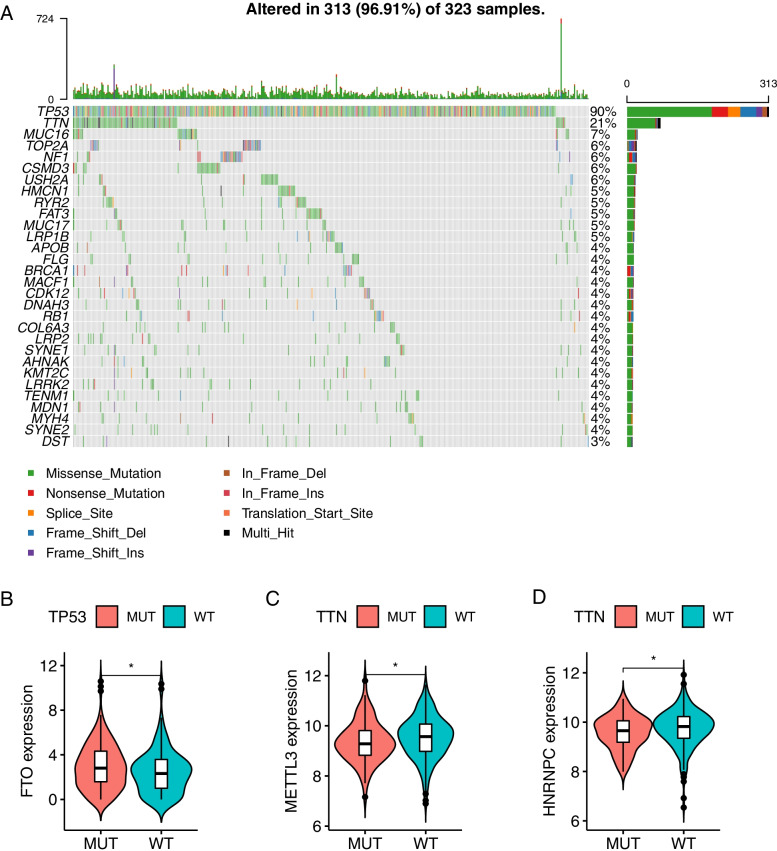
Landscape of genetic of m6A -related genes in ovarian cancer. **A** The mutation frequency of genes in 323 patients with ovarian cancer from TCGA-OV cohort. Each column represented individual patients. The upper barplot showed TMB. The number on the right indicated the mutation frequency in each regulator. The right barplot showed the proportion of each variant type. **B** The correlation between *TP53* mutation and *FTO*. **C-D** Correlation between *TTN* mutation and *HNRNPC* and *METTL3*. The asterisks represented the statistical p value (*P < 0.05)

**Fig. 4:**
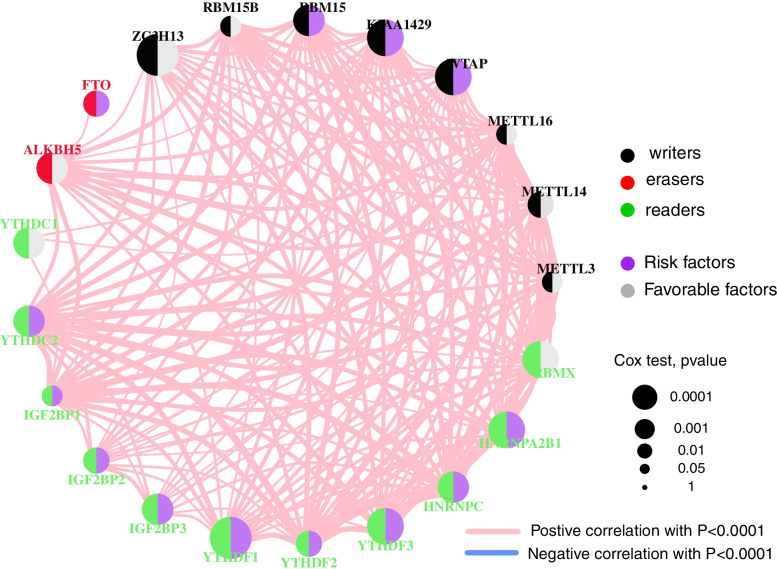
The interaction between m6A-related genes in ovarian cancer. The circle size represented the effect of each regulator on the prognosis, and the range of values calculated by cox test was *P* < 0.0001, *P* < 0.001, *P* < 0.01, *P* < 0.05 and *P* < 1, respectively. Purple part in the circle, risk factors of prognosis; Grey part in the circle, favorable factors of prognosis. The lines linking regulators showed their interactions, and thickness showed the correlation strength between regulators. Negative correlation was marked with blue and positive correlation with red. Writer, erasers and readers were marked with black, red and green, respectively

Using the expression levels of 21 m6a-related genes, samples from the TCGA-OV data set were divided into two groups according to the optimal density algorithm. High expression of *METL3*, *ZC3H13*, *ALKBH5*, and *YTHDC1*, and low expression of *WTAP*, *KIAA1429*, *RBM15*, *YTHDC2*, and *YTHDF1* correlated with good overall survival (OS) prognosis (Fig. 2). Indeed, 96.91% of tumor samples had gene mutations, of which 90% and 21% were in *TP53* and *TTN* respectively (Fig. 3A). Furthermore, mutations in *TP53* were significantly correlated with high expression of *FTO* (*P* < 0.05)(Fig. 3B), and mutations in *TTN* were significantly correlated with low expression of both *METTL3* and *HNRNPC* (*P* < 0.05)(Fig. 3C and D). The expression of 21 m6A-related genes is correlated, indicating that the expression of genes can promote each other (Fig. 4).

### Identification of m6A subtypes and differentially expressed genes in OV

**Fig. 5:**
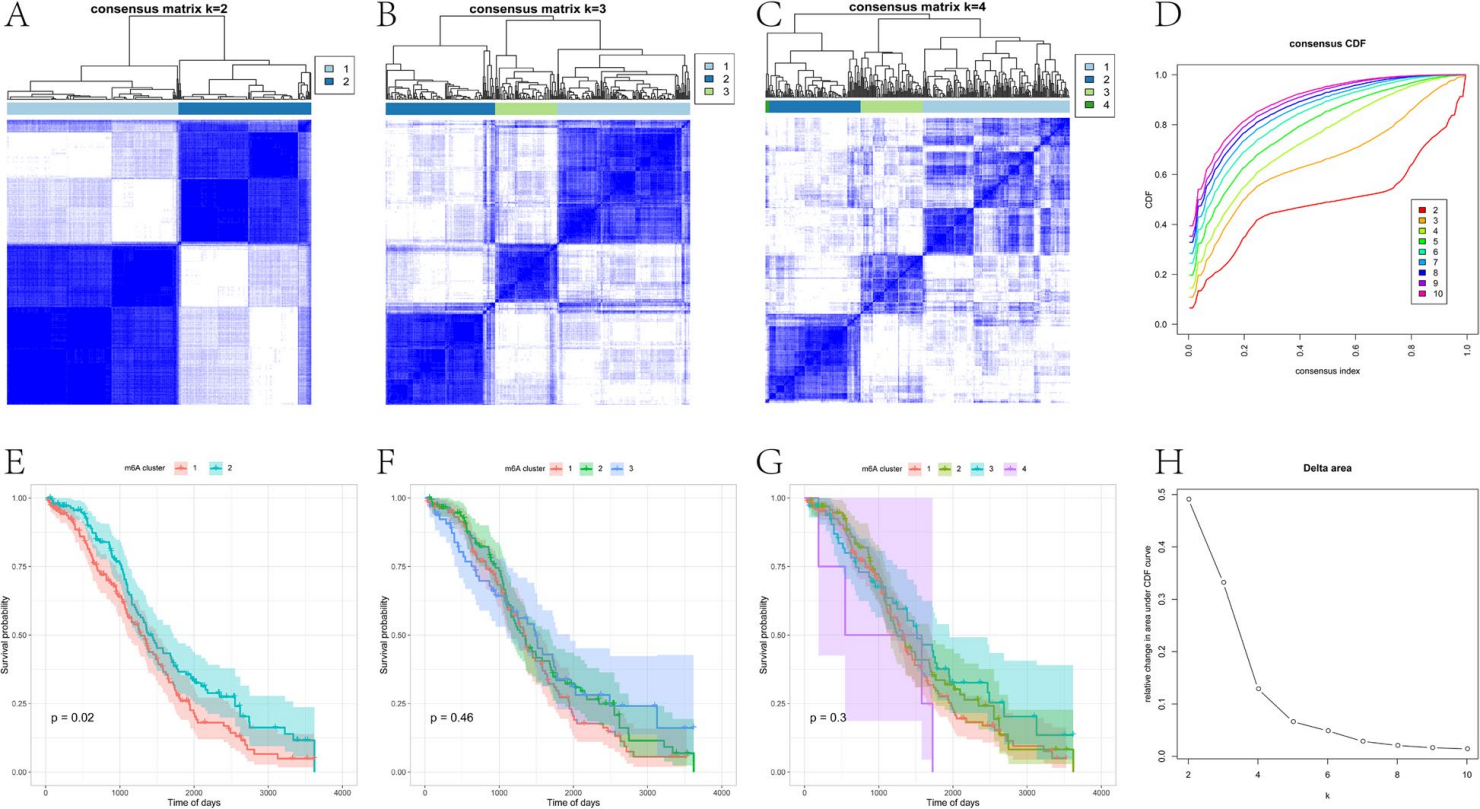
Consensus clustering of tumor m6A-related gene expression profiles. **A-C** The clustering results when the number of classifications is k = 2, 3, and 4. **D** CDF curve distribution of uniform clustering. **E–G** The survival curve when the number of classifications is k = 2, 3, and 4. **H** The distribution of the area under the CDF curve of consistent clustering

Two independent m6A subtypes with obvious survival differences were determined based on the expression values of 21 m6A-related genes. m6A-2 had a significantly better prognosis than m6A-1, with a median survival time of 1,123 days. Conversely, m6A-1 was associated with poor prognosis, with a median survival time of only 941 days (Fig. 5).

**Fig. 6:**
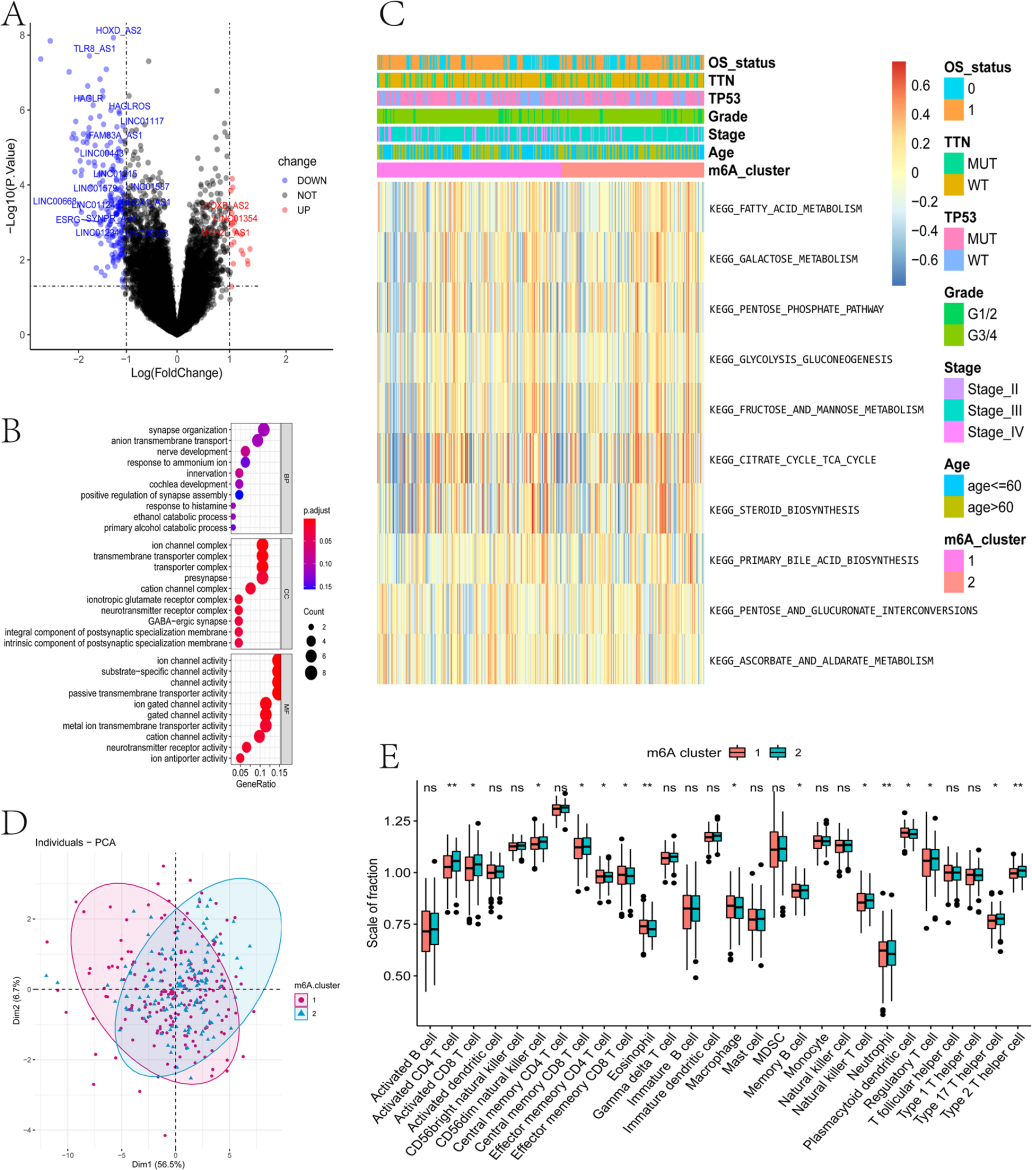
Identification and functional analysis of differentially expressed genes among tumor m6A subtypes. **A** Volcano plots displaying the genes that were differentially expressed between the tumor m6A subtypes. **B** Functional annotation using GO enrichment analysis. The size of the bubble represented the number of genes enriched. **C** Enrichment fraction heat map of the KEGG pathway. The OS status, TTN, TP53, tumor stage, gender, age and m6A clusters were used as patient annotations. **D** PCA analysis of the expression profile. **E** The abundance of each tumor immune infiltrating cell in two m6A subtypes. The upper and lower ends of the boxes represented interquartile range of values. The lines in the boxes represented median value, and black dots showed outliers. The asterisks represented the statistical *P* value (*P < 0.05; **P < 0.01)

We identified the DEGs between the m6A subtypes (TableS1), of which eight were highly expressed in the m6A-1, and 135 genes were highly expressed in m6A-2 (Fig. 6A). GO functional enrichment analysis results are displayed with bubble diagrams (Fig. 6B), and the top ten pathways enriched were in three functional categories: biological process (BP), molecular function (CC), and molecular functions (MF). Most of the enriched pathways were related to biological processes such as synaptic organization, ion channels, and transmembrane transport. Among the identified DEGs, there are 20 lncRNAs (Table S2), and their expression levels are significantly different between m6A-1 and m6A-2 (*P* < 0.05)(Fig. 6D). And the top ten pathways enriched in KEGG enrichment analysis were fatty acid metabolism, galactose metabolism, pentose phosphate pathway, glycolysis gluconeogenesis, fructose and mannose metabolism, citrate cycle, TCA cycle, steroid biosynthesis, primary bile acid biosynthesis, pentose and glucoronate interconversions, and ascorbate and aldarate metabolism (Fig. 6C). The ggGSEA method further analyzed the infiltration patterns of m6A-1 and m6A-2 subtypes in 28 immune cells (Fig. 6E), and there was consistency between the expression profile and prognosis profile of m6A-related genes in OV tumors, indicating accurate classification of the two m6A subtypes.

### Construction of m6A-related lncRNA risk score model for ovarian cancer

**Fig. 7:**
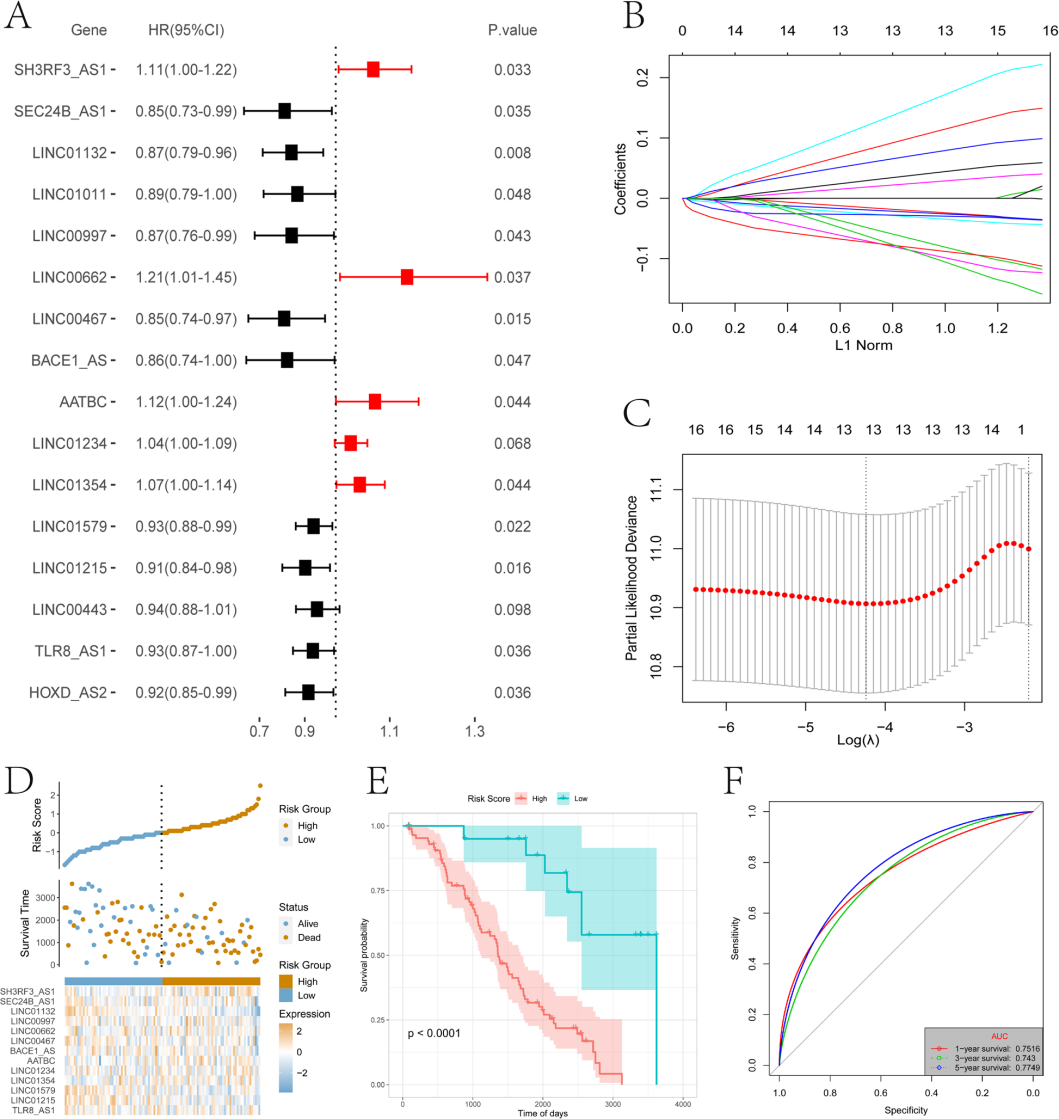
Screening of lncRNA and construction of risk model. **A** Single factor cox regression and forest plot were applied to examine the hazard ratio (HR) and 95% confidence interval of each lncRNA. **B** The change trajectory of each independent variable, the horizontal axis represents the log value of the independent variable lambda, and the vertical axis represents the coefficient of the independent variable. **C** Confidence interval under each lambda. **D** Distribution map of risk score. **E** Overall survival analysis for patients in high/low risk. **F** The ROC curve of risk score

Pearson correlation coefficient indicated that lncRNAs are co-expressed with m6A-related genes (*P*-value < 0.001 and |R|> 0.5), and 361 lncRNAs (Table S3) had a significant co-expression relationship with at least one m6A-related gene. In order to include more m6A-related lncRNAs, we combined 143 DEGs with 361 co-expressed genes, and got 381 m6A-related lncRNAs to construct a risk scores model for tumor immune cell infiltration. First, the TCGA-OV overall set (*n* = 323) was divided into a training set (*n* = 215) and a test set (*n* = 108) according to an approximate 2:1 ratio. In the training set, single factor cox was used to analyze the 381 candidate lncRNAs (*P*-value < 0.1), and only 16 lncRNAs were retained (Table S4, Fig. 7A). For the convenience of clinical use, LASSO further screen the variables and retained 13 lncRNAs (Fig. 7B-C). Finally, multi-factor cox regression constructed a lncRNA risk scoring model related to tumor immune cell infiltration. The final 13-lncRNA gene signature formula is as follows:

$$Risk Score = (-0.065)*TLR8-AS1 + (-0.071)*LINC01215 + (0.009)*LINC01579 + (-0.032)*LINC01354 + (0.057)*LINC01234 + (0.116)*AATBC + (-0.225)*BACE1-AS + (0.052)*LINC00467 + (0.393)*LINC00662 + (-0.102)*LINC00997 + (0.272)*LINC01132 + (-0.503)*SEC24B-AS1 + (-0.015)*SH3RF3-AS1$$

In order to judge the impact of the risk scores constructed by these 13 lncRNAs on OS training samples were divided into high- and low-risk groups (Fig. 7D). The high-risk group had a higher proportion of death samples. Kaplan–Meier analysis showed that the OS of patients in the high-risk group was significantly lower than that of the low-risk group (*P* < 0.05) (Fig. 7E). Risk scores accurately predicted the OS of the TCGA-OV data set. The one-, three-, and five-year areas under the curves were 0.7516, 0.7430, and 0.7749, respectively (Fig. 7F).

**Fig. 8:**
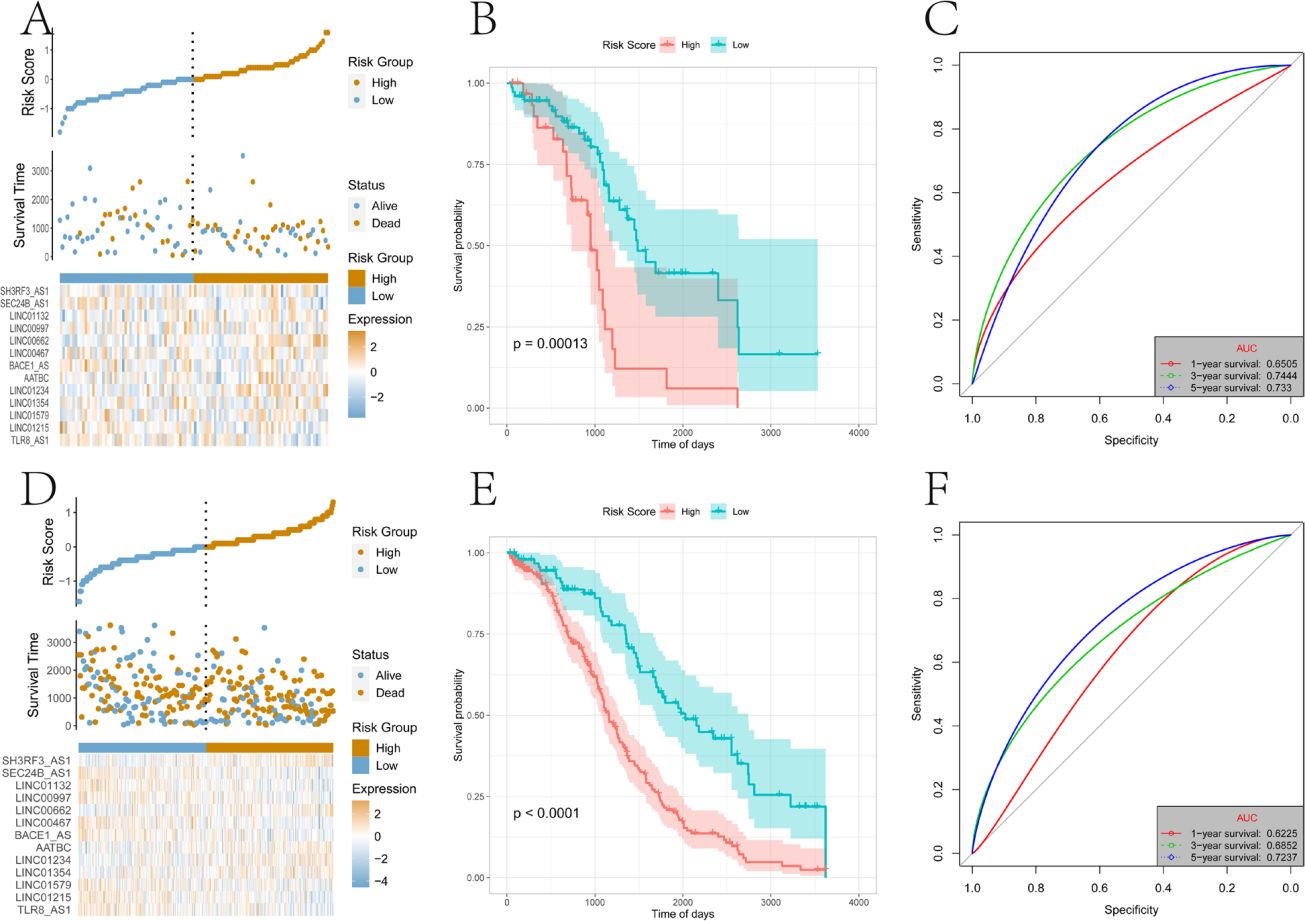
Test set and overall set to verify the risk model. **A** The distribution map of the risk score of the test set. **B** Survival curve of the test set. **C** The ROC curve of the test set at one-, three-, and five-years. **D** Distribution map of the overall set of risk scores. **E** Overall set survival curve. **F** The ROC curve of the overall set of one-, three-, and five-years

Subsequently, similar processing was applied to the test set and the overall set of TCGA-OV and samples were divided into high- and low-risk groups. Again, the high-risk group had a higher proportion of death samples (Fig. 8A, 8D). Kaplan–Meier analysis showed that OS of patients in the high-risk group was significantly lower than that of the low-risk group (*P* < 0.05)(Fig. 8B, 8E). Risk scores accurately predicted OS in the TCGA-OV test set and overall data set. The test set’s one-, three-, and five-year AUCs were 0.6505, 0.7444, and 0.7330, respectively (Fig. 8C), and the overall set’s one-, three-, and five-year AUCs were 0.6225, 0.6852,and 0.7237, respectively (Fig. 8F).

**Fig. 9:**
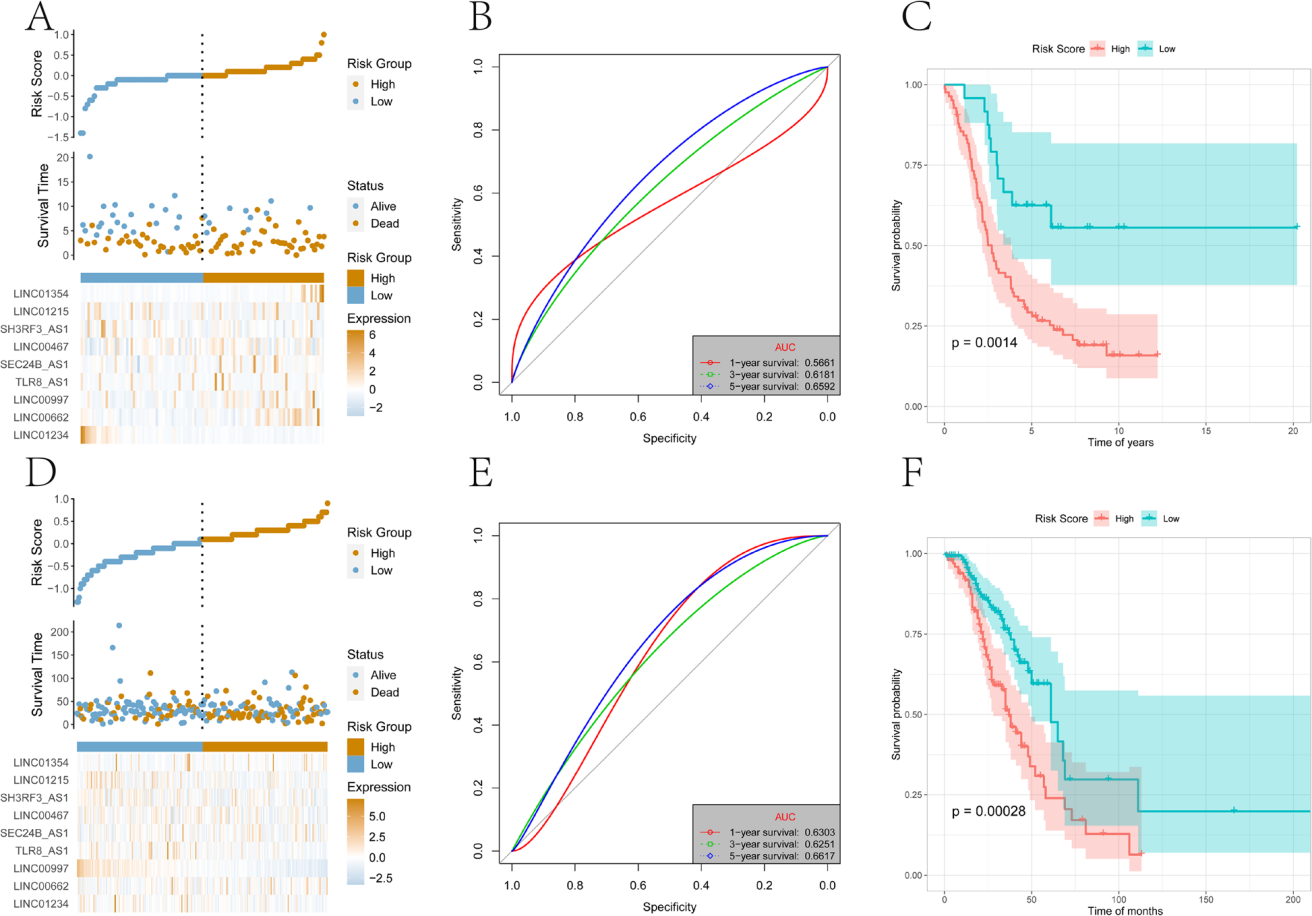
The external data sets GSE26193 and GSE9891 validate the risk model. **A** The distribution map of the risk score of the GSE26193 data set. **B** ROC curve of GSE26193 data set at one-, three-, and five-years. **C** Survival curve of the GSE26193 data set. **D** The distribution map of the risk score of the GSE9891 data set. **E** ROC curve of the GSE9891 data set at one-, three-, and five-years. **F** Survival curve of the GSE9891 data set

In order to further evaluate the robustness of the risk scores constructed by 13-lncRNA in predicting the OS of OV tumors, this study selected two data sets, GSE26193 and GSE9891, from the GEO database for analysis. First, samples were divided into high-and low-risk groups, and the high-risk group had a higher proportion of death samples (Fig. 9A, 9D). Kaplan–Meier analysis showed that the OS of patients in the high-risk group was significantly lower than that of the low-risk group (*P* < 0.05)(Fig. 9B, 9E). As shown in the GSE26193 data set, the risk scores value accurately predicted OS, and its one-, three-, and five-year AUCs were 0.5661, 0.6181, and 0.6592 respectively (Fig. 9C). Similarly, in the GSE9891 data set, the risk scores value also accurately predicted OS, and its one-, three-, and five-year AUCs were 0.6303, 0.6251, and 0.6617 respectively (Fig. 9F).

### The relationship between risk score and clinical features

**Fig. 10:**
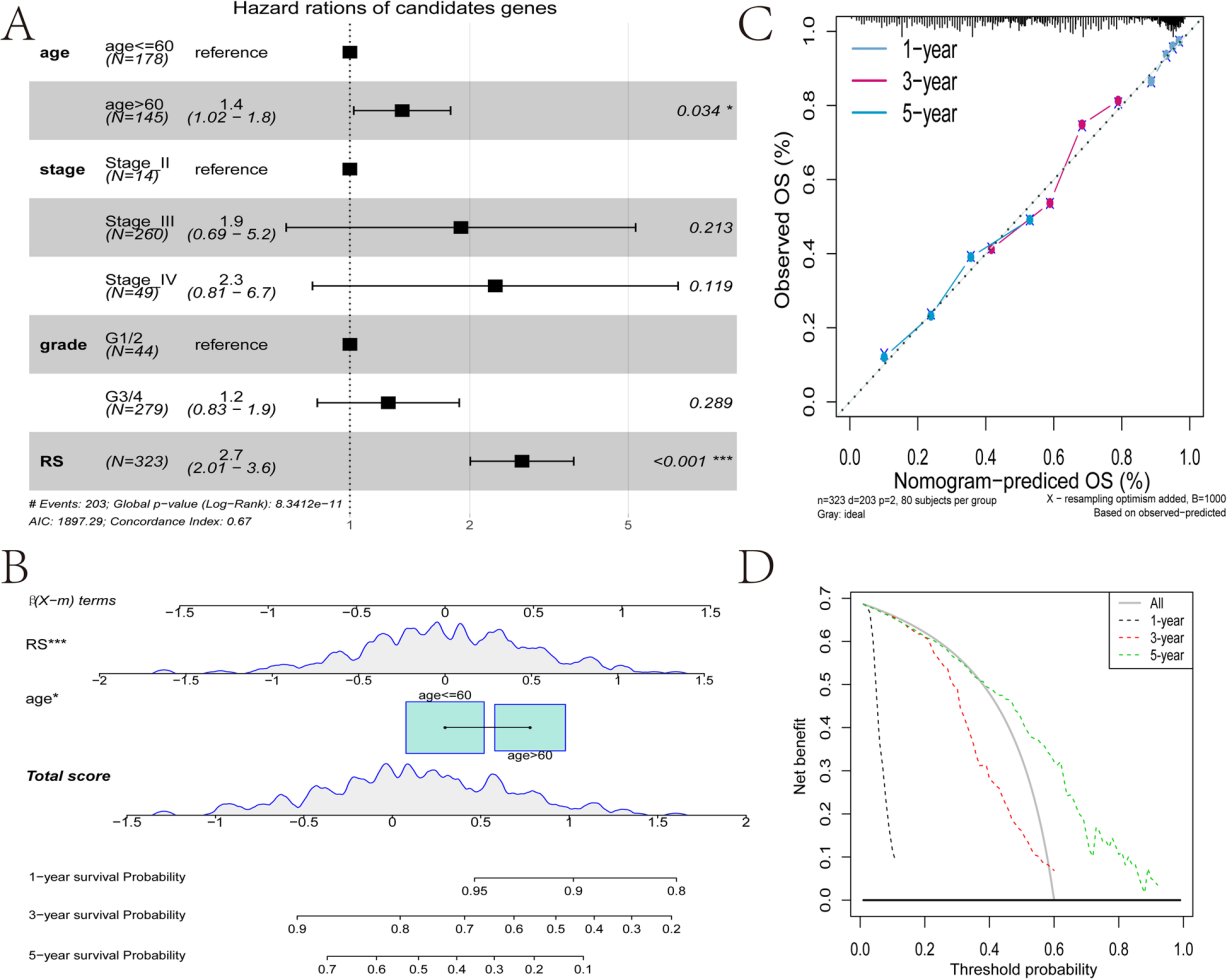
The clinical value of predictive models. **A** Forest plot of multivariate cox analysis. **B** Nomogram predicts patients' OS at one-, three- and five-years. **C** The calibration curve of the nomogram predicts the patient's one-, three-, and five-year OS relative to the actual survival time. **D** The DCA curve evaluates the clinical benefits and the application range of the nomograms. Black indicates all samples are negative and have not undergone any treatment, so the net profit is 0. Gray indicates that all samples are positive and have been processed. The x-axis represents the threshold probability of patients with the condition

Patient age and tumor grade are important clinical characteristics, consequently, it is necessary to clarify their relationship with tumor risk score. First, multivariate cox analysis determined that risk score is an independent prognostic factor different from age, stage, and grade (Fig. 10A). Next, we used risk score and age, since were both significant independent prognostic indicators, to construct a nomogram for the convenience of clinical judgment. The result show that Risk score had a more significant effect on prognosis (*P* < 0.05)(Fig. 10B). The calibration curve showed that the nomogram had high accuracy (Fig. 10C). The DCA curve analysis showed that the net benefit in the nomogram when predicting the five-year survival was higher than at one- and three-years, meaning the model is more suitable for predicting the five-year patient survival (Fig. 10D).

### Relationship between tumor risk score and tumor mutation burden

**Fig. 11:**
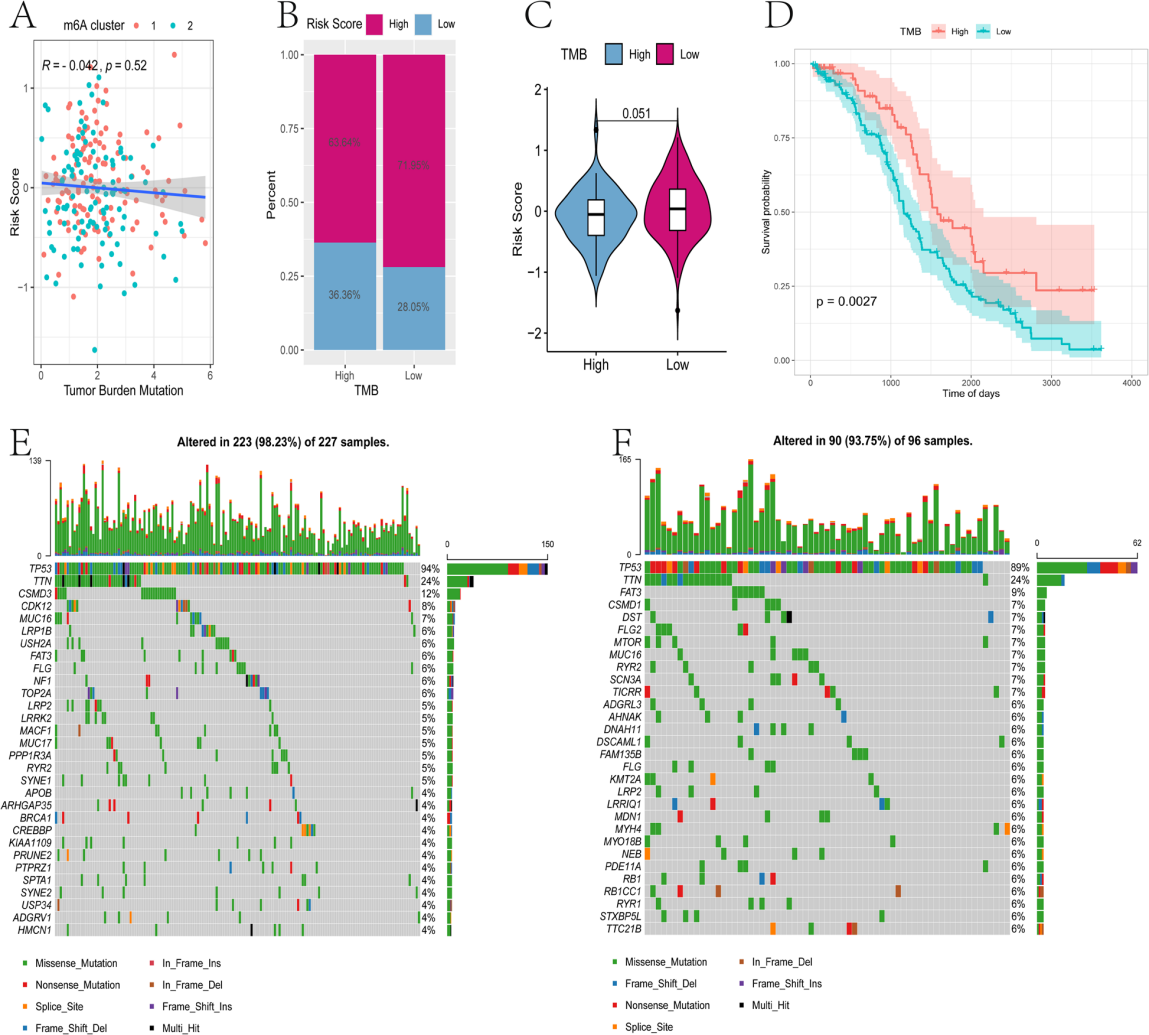
The relationship between tumor risk score and tumor mutation burden. **A** Correlation linear regression analysis shows that risk score is significantly negatively correlated with TMB. **B,C** Proportional distribution bar graph and Violin diagram of the relative distribution of TMB in high risk score versus low risk score subgroups. **D** Survival curve of high- and low-TMB score groups. **E** Waterfall chart of gene mutations in the high-risk group. **F** Waterfall chart of gene mutations in the low-risk group

Tumor mutational burden (TMB) may determine an individual's response to cancer immunotherapy, therefore exploring the relationship between TMB and risk score may clarify the genetic characteristics of each m6A subgroup. We used the Survminer package in R to calculate the optimal density gradient threshold associated with the TMB score and patient survival. As previously, the tumor samples in TCGA-OV were divided into high- and low-TMB score groups, and there was a significant difference in survival between the two groups (Fig. 11D). Subsequently, a correlation analysis was performed and risk score was significantly negatively correlated with TMB (*P* < 0.05)(Fig. 11A). Furthermore, when comparing the TMB of patients between the high- and low-risk score groups, TMB of the high-risk group was significantly lower than the low-risk group (Fig. 11B and C).

Next, we evaluated the distribution of somatic variation in OV driver genes between the high- and low-risk groups, and compared the top 30 driver genes with the highest change frequency (Fig. 11E and F). By analyzing the mutation annotation files of the TCGA-OV cohort, there were significant differences in the mutation profiles between the high and low immune cell infiltration (ICI) subgroups (*P* < 0.05). These results may provide new ideas for studying the mechanism of tumor m6A status and gene mutations in immune checkpoints.

### The relationship between risk score and immune cell infiltration

**Fig. 12:**
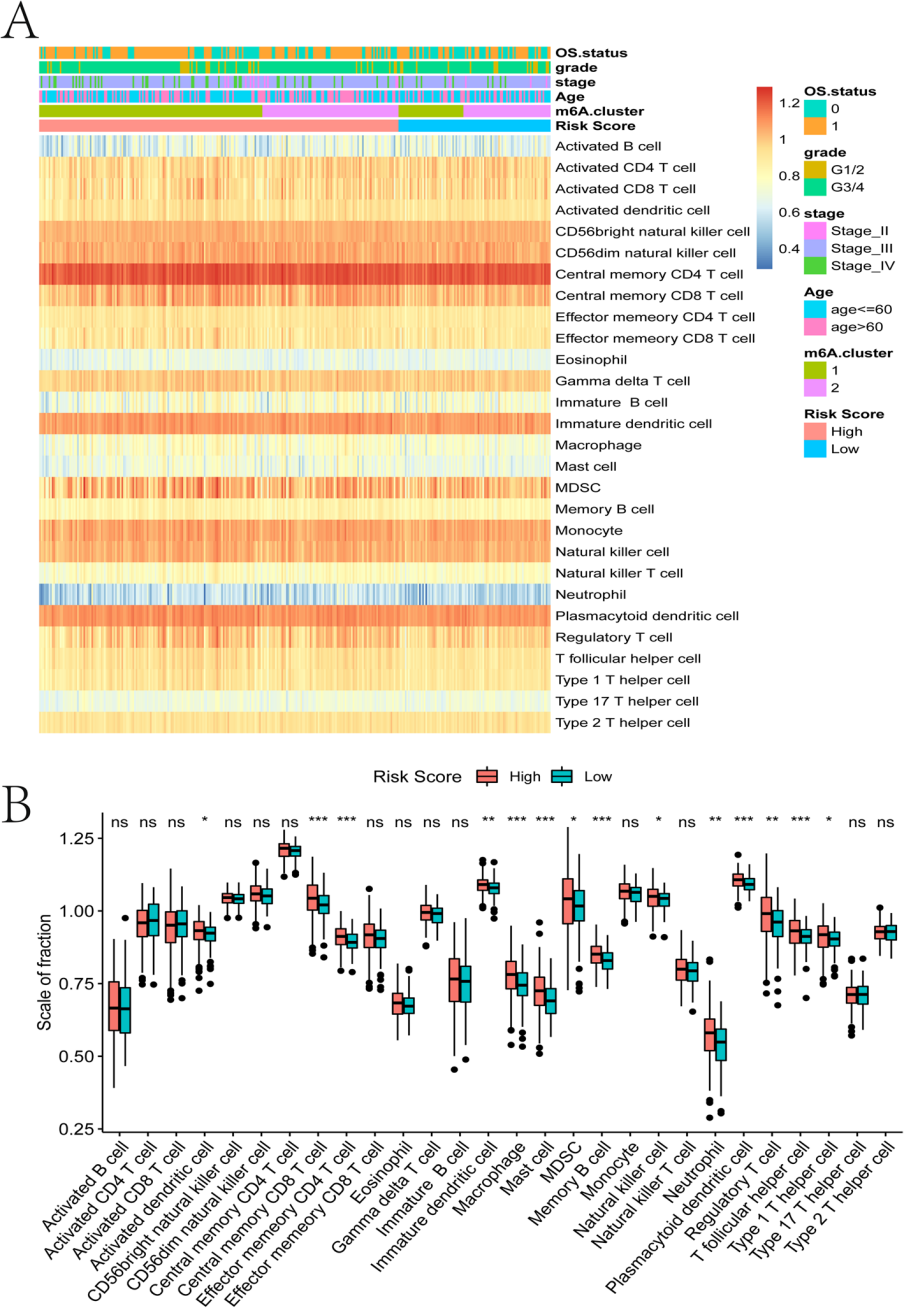
The relationship between tumor risk score and immune cell infiltration. **A** Heat map of the distribution of immune cell infiltration ratio. **B** Box plot of differences in immune cell infiltration between high and low risk score groups

In order to explore the relationship between the risk score constructed by tumor m6A-related lncRNAs and the tumor immune microenvironment, we used GSEA to evaluate the tumor infiltration status of 28 immune cells in the TCGA-OV data set. From the overall level of tumor immune cell infiltration, immune cells with high levels of infiltration in OV tumors were central memory CD4 T cells, CD56 bright natural killer cells, CD56 dim natural killer cells, immature dendritic cells, monocytes, natural killer cells, and plasmacytoid dendritic cells. The overall low-level infiltrating immune cells are mainly neutrophils (Fig. 12A). Next, a hypothesis test was performed on the difference in immune cell infiltration in the high and low risk score groups, and the results showed that the high-risk group had significantly higher infiltration levels of activated dendritic cells, central memory CD8 T cells, effector memory CD4 T cells, immature dendritic cells, macrophages, mast cells, MDSCs, memory B cells, natural killer cells, neutrophils, plasmacytoid dendritic cells, regulatory T cells, T follicular helper cells, and type 1 T helper cells than the low-risk group (Fig. 12B).

### Assess the ability of tumor risk score to predict the benefit of immunotherapy

**Fig. 13:**
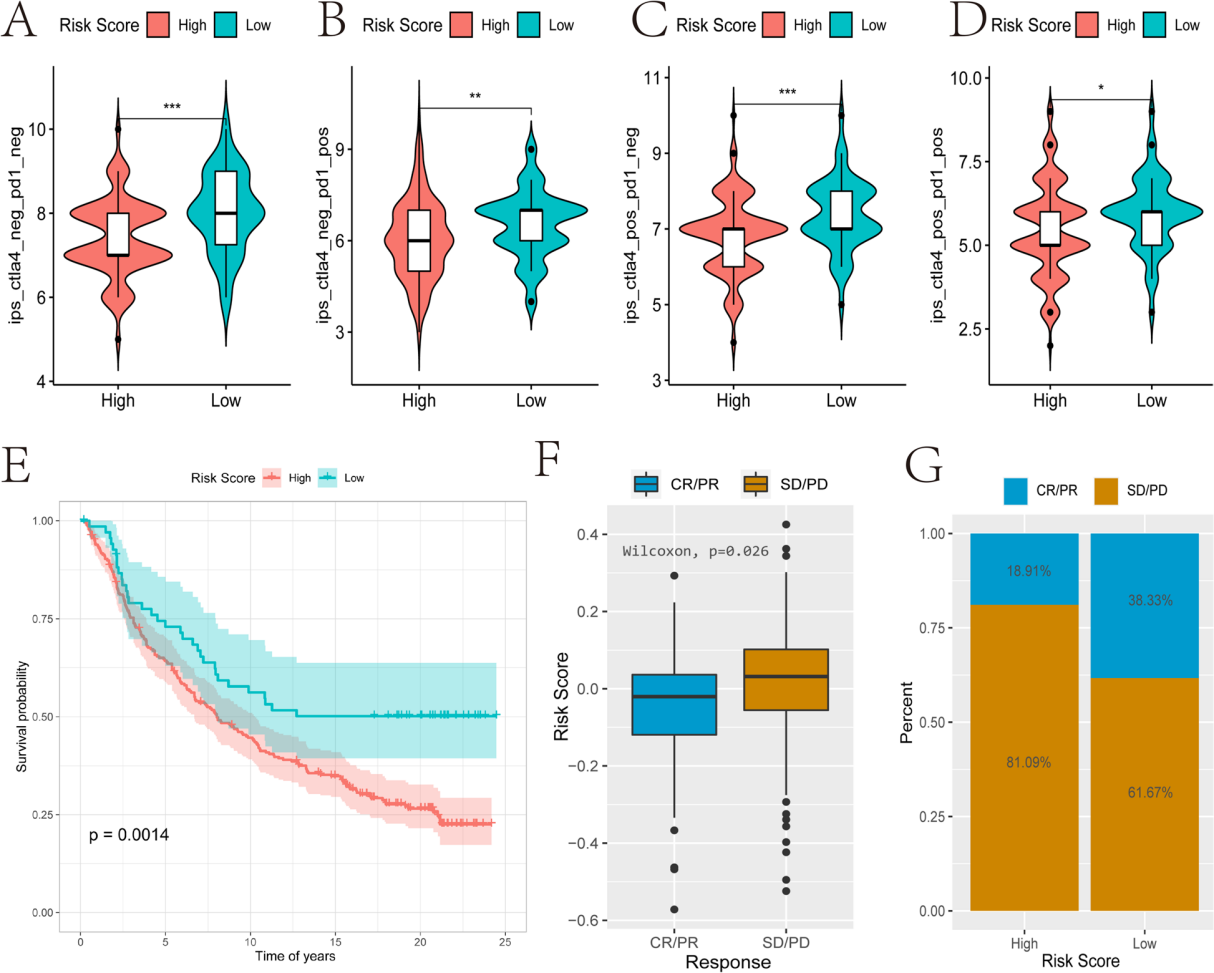
The relationship between tumor risk score and immunotherapy. **A-D** The correlation between risk score and different immune scores. **E** The prognostic difference between the high and low risk score groups. **F-G** The difference in risk scores between the remission group (CR/PR) and the non-remission group (SD/PD). CR, complete response; PR, partial response; SD, stable disease; PD, progressive disease

In order to explore the predictive ability of risk score in the benefit of patients with immunotherapy, this study was based on the IPS score of the TCGA-OV sample in the TCIA database and the IMvigor210 data set (http://researchpub.gene.com/IMvigor210CoreBiologies) of the immunotherapy cohort to perform related evaluation and analysis. Immunophenoscore (IPS) score can determine the immunogenicity of tumors. The IPS scores of the four types (ips_ctla4_neg_pd1_neg, ips_ctla4_pos_pd1_neg, ips_ctla4_neg_pd1_pos, and ips_ctla4_pos_pd1_pos) against risk score and found that all four were significantly higher in the low-risk group than the high-risk group, suggesting that patients in the low-risk group are more likely to benefit from immunotherapy. In the IMvigor210 cohort, patients receiving anti-PD-L1 immunotherapy were assigned high- or low-risk scores, with patients in the low-risk group having a better prognosis than those in the high-risk group (Fig. 13E). Risk score then, is related to the objective response of anti-PD-L1 treatment (Fig. 13F). The non-remission group (SD/PD) was associated with a higher risk score than the remission group (CR/PR) (Fig. 13G, 81.09% vs. 61.67%). Complete remission (CR): All target lesions disappeared, no new lesions appeared, and tumor markers remained normal for at least four weeks. Partial remission (PR): The total maximum diameter of the target lesion is reduced by ≥ 30%, and it is maintained for at least four weeks. Stable disease (SD): The sum of the maximum diameters of the target lesions, the reduction does not reach the PR, or the enlargement does not reach the PD. Progressive disease (PD): The sum of the maximum diameters of the target lesions increased by at least ≥ 20%, or new lesions appeared. CR + PR = objective relief (OR). Reduced less than PR (baseline lesion total length diameter reduction ≥ 30%) or increased less than PD (baseline lesion total length diameter increased ≥ 20% or new lesions appeared, or/and non-target lesions progressed), one or more non-target lesions and/or abnormal markers.). Overall, this indicated that the risk score constructed by the m6A-related lncRNA model may be related to immunotherapy response.

## Discussion

OV is one of the three major malignant tumors in gynecology. Due to early asymptomatic tumor metastasis and treatment resistance of ovarian cancer, the five-year survival rate of patients is very low [22]. At present, there is no effective way to predict OV prognosis. It has been found that the invasion and metastasis of OV is regulated by the molecular characteristics of the genome [23]. In various cancers, writers catalyze m6A modification in the mRNA of oncogenes or tumor suppressor genes, and then readers recognize the behavior of these markers through a series of molecular biological effects, thereby up-regulating or down-regulating the expression of oncogene and tumor suppressor gene. Conversely, erasers remove m6A from the mRNA to cause the modification, preventing readers from recognizing and playing its molecular biological roles, thereby up-regulating or down-regulating the expression of oncogene and tumor suppressor gene[24]. Some m6A regulators are abnormally expressed in OC, such as YTHDF1, YTHDF2, METTL3, ALKBH5 etc., and promote or disrupt the development or maintenance of tumour phenotypes [25]. As the most common mRNA and lncRNA modification, m6A methylation plays different roles in a variety of tumors, including occurrence, proliferation, invasion, and metastasis [3, 24, 26]. At present, the role of m6A modification in the prognosis of OV is still unclear. We divided 21 m6A RNA methylation-related genes into two groups, high- and low-expression, and found that expression of most of these genes is related to prognosis (Fig. 2). Indeed, *YTHDF1* is often amplified in ovarian cancer, and its up-regulation is related to poor prognosis [27]. *METTL3* predicts shorter overall survival in glioblastoma, longer survival in breast cancer [28], and is also highly expressed in OV and promotes cell proliferation, lesion formation, movement, invasion, and tumor formation, and indicates poor prognosis [29]. High expression of *ALKBH5* in OV also indicates poor prognosis [30]. However, most studies focus on a single m6A-related gene, making it difficult to elucidate the overall prognostic predictive effect mediated by the integration of multiple m6A modulators. We found that expression levels of 21 m6A-related genes are related to and promote the expression of each other (Fig. 4), so there may be a synergistic effect on the predicted results.

Tumor cells continue to mutate as they progress, which is reflected not only the genetics, but also antigen expression, cell morphology, and behavior [31]. Tumor heterogeneity allows tumors to resist external pressure, and is closely related to tumor metastasis, drug resistance, and clinical prognosis [32]. We found that 96.91% of OV samples have genetic mutations, most significantly in *TP53* and *TTN*, which were significantly related to the expression of m6A-related genes *FTO*, *METTL3*, and *HNRNPC* (Fig. 3). *TP53* is a tumor suppressor gene highly associated with tumors [33]. After mutation, *TP53* has a gain of function (GOF), including inhibiting the activity of wild-type *TP53* and inducing abnormal gene expression to promote tumorigenesis. Epithelial ovarian cancer has a high degree of genetic instability characterized by *TP53* mutations [34, 35]. *FTO* expression is significantly downregulated in thyroid cancer, as a tumor suppressor, and may affect metastasis through the *TP53* pathway [36]. TTN (titin) is a structural protein in striated muscle, and plays a role in protein formation in muscle fibers, maintains static tension and elongated elasticity [37]. TTN, which is frequently detected in solid tumors, is associated with increased TMB and correlated with objective response to Immune checkpoint blockade [38]. Study found that TP53 and TTN mutations were associated with the expression of UBE2T, which was associated with poor prognosis of ovarian cancer [39]. The mutations in *TP53* and *TTN* in OV are inseparable from tumor heterogeneity. We found that m6A-related genes are also involved in the mutation process, indicating that m6A may play a role in tumor heterogeneity.

We typed m6A-related genes based on prognostic analysis (Fig. 5). Analyzing DEGs, the GO function enrichment of highly expressed genes is mainly in the processes of synaptic tissue, ion channels and transmembrane transport, while KEGG enrichment is related to energy conversion and metabolism (Fig. 6). In order to adapt to rapid proliferation and differentiation, the information transmission and material transformation between or within tumor cells must increase significantly, and energy conversion needs to be reconstructed, creating a microenvironment suitable for tumor cell survival, enhancing the ability of invasion and metastasis, and also helping tumor cells to escape the body’s immune system and apoptosis mechanisms [40]. m6A-related genes are closely related to tumor metabolism [41]. The m6A modification on lncDpf3 in dendritic cells regulates the recognition and binding of YTHDF2, thereby inhibiting the glycolytic metabolism regulated by the glycolytic gene Ldha [42].

We also found that the ICI pattern was significantly related to the m6A subtype (Fig. 6). The m6A-1 subtype was characterized by poor prognosis with a significant increase in effector memory CD8T cells, macrophages, and plasmacytoid dendritic cells, and a significant decrease in activated CD4 T cells and activated CD8 T cells. In tumor immunity, tumor ICI is the result of mutual balance and mutual adaptation with the tumor cell [43]. At the same time, it is also a competition between the body's anti-tumor immune response and tumor immune escape, which affects the occurrence of tumors and the regulation of the body's immune system [43]. These results indicate that there is a potential relationship between m6A and ICI, and indeed, our risk model verified the relationship between m6A-related genes, prognosis, and the immune microenvironment.

lncRNA not only regulates the proliferation, differentiation, invasion, and metastasis of cancer cells, but also regulates metabolic reprogramming [44, 45]. lncRNA promotes energy metabolism and cancer progression through post-translational modifications including ubiquitination, phosphorylation, and acetylation of key metabolism-related proteins [46, 47]. lncRNA is closely related to tumor progression and plays an important role in the malignant transformation of OV [48, 49]. lncRNAs such as NBAT-1 and RP11-190D6.2 are down-regulated in ovarian cancer cells and are significantly correlated with FIGO stage and tumor size [50, 51]. lncRNAs such as lncBRM, LINC00152, and EIBC are up-regulated in ovarian cancer, and are also related to FIGO stage, histological classification, lymph node metastasis, and poor prognosis [52–54]. Based on the GSE9891 and GSE30161OC microarray data sets extracted from the GEO, six lncRNAs were related to the recurrence of OV [55]. Therefore, the abnormal expression of specific lncRNAs can be used as independent biomarkers for the diagnosis and prognosis of OV and effectively predict tumor progression.

Screening of the differentially expressed lncRNAs in m6A and the lncRNA sets co-expressed with m6A-related genes, yielded 13 major lncRNAs (TLR8-AS1, LINC01215, LINC01579, LINC01354, LINC01234, AATBC, BACE1-AS, LINC00467, LINC00662, LINC00997, LINC01132, SEC24B-AS1, SH3RF3-AS1) to construct the lncRNA risk score model related to prognosis and tumor ICI. TLR8-AS1 enhances the metastasis and chemotherapy resistance of ovarian cancer cells in vivo and in vitro and high expression is associated with poor prognosis [56]. LINC01354 is significantly increased in non-small cell lung carcinoma, promotes the proliferation and invasion of lung cancer cells, and high expression is related to advanced TNM stage and poor prognosis [57]. LINC01234 is upregulated in colorectal cancer, gastric cancer, and oral cancer and high expression correlates with poor prognosis. It is related to tumor stage and lymph node metastasis, and promotes cancer cell proliferation, metastasis, and inhibits apoptosis [58–60]. BACE1-AS is significantly overexpressed in liver cancer, has a tumorigenic effect, and predicts poor overall survival and recurrence-free survival[61]. LINC00467 is significantly upregulated in non-small cell lung cancer, promotes tumor cell growth and metastasis, and is related to clinical stage and poor prognosis [62]. It also promotes the proliferation, migration, invasion, and epithelial-mesenchymal transition (EMT) of breast cancer cells, tumor growth and lung metastasis in vivo, and high expression predicts poor OS [63]. In renal clear cell carcinoma, higher levels of LINC00997 are associated with lower OS and disease-free survival [64]. Overexpression of LINC01132 in OV is significantly related to the poor prognosis, and it promotes the proliferation, migration, invasion, inhibition of apoptosis, and tumor growth in vivo [65]. Clearly, most of the lncRNAs in the risk model are involved in predicting the prognosis of different tumors, so it is unsurprising that they were included in the model. Indeed, the integration of these lncRNAs may have a better synergistic effect in predicting the prognosis of OV and the effect of immunotherapy together rather than individually. Consistent with expectations, the risk scores in the training set, test set, and overall set showed very accurate prediction capabilities (Fig. 7 and 8), and the robustness of the model was further verified by the GEO data set (Fig. 9). Together with clinicopathological parameters, risk scores had strong independent predictive ability, especially for long-term survival (Fig. 10).

Many m6A mRNA abnormalities are found in different immune cells in tumors. Compared with wild-type, in *YTHDF1*-/- mice, due to the enhanced ability of dendritic cells to present tumor neoantigens, the antigen-specific anti-tumor response mediated by CD8 + T cells is enhanced [66]. The m6A methylation of *METTL3*/*FTO* promotes the generation and polarization of M1/M2 macrophages from bone marrow-derived macrophages (BMDM) [67, 68]. Dendritic cells knocked out of *METL3* have a reduced ability to stimulate differentiation and activation of CD4 + T cells in vivo [69]. *METTL3*-/- CD4 + regulatory T cells (Tregs) show systemic loss of function and are unable to stimulate naive T cell proliferation [66]. In order to explore the relationship between risk score constructed by m6A-related lncRNAs and the tumor immune microenvironment, 28 different ICI states were evaluated, and in the high-risk group, immune cells such as activated dendritic cells, central memory CD8 T cells, macrophages, and MDSCs were more abundant (Fig. 12). Immunosuppression is an important feature in the tumor microenvironment and is characterized by depleted killer immune cells and antigen presenting cells (APC) and suppressive immune cells recruited or induced at a high levels, such as Tregs, tumor-associated macrophages (TAMs), immature myeloid cells (iMC)/myeloid-derived suppressor cells (MDSCs) and a variety of cytokines [70]. In the process of tumor progression, in the abnormal tumor microenvironment, a variety of immune cells that are recruited are remodeled, activating new functions, and helping tumor cells avoid elimination, and promoting further invasion and metastasis [71, 72].

Increasingly immunotherapies targeting immune checkpoints (ICB, PD-1/L1, CTLA-4, etc.) have surprising effects in long-term responses in a small number of patients, however, there is little clinical benefit in the majority of patients [73]. Tumor progression is a complex process, including cytogenetic and epigenetic variations[74]. TMB refers to the distribution density of non-synonymous mutations in somatic genes, that is, the total number of coding errors, base substitutions, gene insertions or deletions per Mb base in the exon coding region [75], and can be used as a biomarker for immune checkpoint inhibitors and to predict the effect of immunotherapy [76]. Tumors with a higher mutation burden can recruit more new antigens to the surface of tumor cells, increase the immunogenicity of the tumor, and thus improve the efficacy of immunotherapy [77, 78]. As found in this study, when comparing the top 30 ovarian cancer driver genes with mutations, there are significant differences in the mutation spectrum between high and low immune cell infiltration (ICI) subgroups and immune infiltration (Fig. 11). In gastric cancer, the m6A modification is significantly associated with tumor mutation burden/microsatellite instability (TMB/MSI) status[79]. After knocking down *METL14*/*YTHDF1*, the transcription levels of IFN-α, -β and -γ, which are essential for tumor cell suppression and anti-tumor immune stimulation, are downregulated [79]. Our data show that there is a significant negative correlation between m6A-related risk score and TMB in OV. Indeed, patients with high TMB status have long-term effects of anti-PD-1/PD-L1 immunotherapy [76]. Based on the IPS score of the TCIA database and the analysis of the IMvigor210 dataset of the anti-PD-L1 immunotherapy cohort, we found that risk score is related to the objective response to anti-PD-L1 therapy, and patients in the low-risk group are more likely to benefit from immunotherapy and get a better prognosis (Fig. 13). This indicates that the m6A-related lncRNA risk score model can accurately predict prognosis and immunotherapy.

This study also has limitations. We used differential expression and co-expressed lncRNAs of 21 m6A-related genes, but new m6A-related genes and new lncRNAs and/or more clinical factors would improve the accuracy of the assessment. We used retrospective data set analysis to establish a risk score, which consequently requires a prospective cohort for verification.

## Conclusion

In this study, we found that the expression profile of m6A-related genes in OV has individual heterogeneity, related to OS, and gene mutations can affect the expression of m6A-related genes. In addition, the risk score model constructed by the differentially expressed and co-expressed lncRNAs related to m6A-related genes can better predict the OS of OV tumor samples and effectively guide immunotherapy strategies.

## Supplementary Information

**Additional file 1.**

**Additional file 2.**

**Additional file 3.**

**Additional file 4.**

## Data Availability

The original contributions presented in the study are included in the article/supplementary material, further inquiries can be directed to the corresponding author.

## Abbreviations

APC: Antigen presenting cells
BP: Biological process
CC: Molecular function
CR: Complete response
DEG: Differentially expressed genes
EMT: Epithelial-mesenchymal transition
FIGO: International Federation of Gynecology and Obstetrics
GEO: Gene Expression Omnibus
GO: Gene Ontology
GOF: Gain of function
GSEA: Gene Set Enrichment Analysis
ICI: Immune cell infiltration
iMC: Immature myeloid cells
IPS: Immunophenoscore
KEGG: Kyoto Encyclopedia of Genes and Genomes
LASSO: Least absolute shrinkage and selection operator
lncRNA: Long-chain non-coding RNA
m6A: N6-methyladenosine
MDSCs: Myeloid-derived suppressor cells
MF: Molecular functions
MSI: Myeloid-derived suppressor cells
MSIGDB: Molecular Signatures Database
OR: Objective relief
OS: Overall surviva
OV: Ovarian cancer
PD: Progressive disease
PR: Partial response
SD: Stable disease
TAMs: Tumor-associated macrophages
TCGA: Using the Cancer Genome Atlas
TMB: Tumor mutation burden
